# Management of Retropharyngeal Lymph Node Metastases in Thyroid Cancer

**DOI:** 10.1002/hed.70027

**Published:** 2025-09-01

**Authors:** Isabelle Fournier, Ashwini Venkata Bandi, Sarah Hamidi, Priyanka Iyer, Naifa L. Busaidy, Maria E. Cabanillas, Ramona Dadu, Mimi I. Hu, Steven G. Waguespack, Ugur Nur Basmaci, Michelle D. Williams, Mena Mansour, Maria Gule Monroe, Neil D. Gross, Ryan P. Goepfert, Nancy D. Perrier, Victoria Banuchi, Jennifer R. Wang, Anastasios Maniakas, Mark E. Zafereo

**Affiliations:** ^1^ Department of Head & Neck Surgery University of Texas MD Anderson Cancer Center Houston Texas USA; ^2^ Department of Endocrine Neoplasia and Hormonal Disorders University of Texas MD Anderson Cancer Center Houston Texas USA; ^3^ Department of Anatomical Pathology University of Texas MD Anderson Cancer Center Houston Texas USA; ^4^ Department of Neuroradiology University of Texas MD Anderson Cancer Center Houston Texas USA; ^5^ Department of Surgical Oncology University of Texas MD Anderson Cancer Center Houston Texas USA

**Keywords:** medullary, papillary, retropharyngeal lymph node metastases, surgery, thyroid cancer

## Abstract

**Background:**

Retropharyngeal lymph node (RPLN) metastases in thyroid cancer are rare, with optimal management underreported.

**Methods:**

Retrospective study of consecutive thyroid cancer patients with RPLN metastases treated at MD Anderson Cancer Center between 2000 and 2024.

**Results:**

One hundred and sixty‐seven patients (75% differentiated, 21% medullary, 4% poorly differentiated thyroid cancer) were divided into three groups: active surveillance (AS) (13%), surgery (56%), and nonsurgical treatment (31%). In the AS group (median follow‐up 2.3 years), RPLN metastases grew a median 3% (range: 0–56) or 0.02 cm (range: 0–0.7) per year. Surgical therapy included transcervical (73%), transoral robotic (14%), transoral (12%), and transmandibular (1%) approaches. Median RPLN metastasis size was 1.7 cm (interquartile range: 1.3–2.2) at surgery. Three months post‐operatively, 11% had dysphagia and 1% had velopharyngeal insufficiency. Nonsurgical treatments included radioactive iodine (10%), radiation therapy (11%), and systemic targeted therapy (79%).

**Conclusion:**

RPLN metastases grow slowly, and those ≥ 1 cm typically undergo surgical resection without significant long‐term morbidity.

## 1 Introduction

The retropharyngeal space (RPS) is divided into suprahyoid and infrahyoid compartments [1]. The suprahyoid RPS is anatomically bounded by the skull base superiorly, the inferior border of the hyoid bone inferiorly, the pharyngeal constrictor muscle anteriorly, the prevertebral fascia posteriorly, and the carotid sheath laterally [1]. The suprahyoid RPS contains both fat and lymph nodes, which are classified into two groups: medial and lateral (or the nodes of Rouvière) [1, 2]. Normal medial retropharyngeal lymph nodes (RPLNs) are generally too small to be detected on imaging, meaning persistent radiographically apparent medial RPLNs are often neoplastic [2, 3, 4]. Lateral RPLNs visible on imaging may be either benign or neoplastic. Neoplastic RPLNs include lymphoma or metastases originating from head and neck cancers, including the nasopharynx [5], oropharynx [6], hypopharynx [7, 8], cervical esophagus [9], and thyroid [10, 11, 12, 13, 14].

Although regional lymph node metastases are present in approximately 30% of patients with thyroid cancer at the time of diagnosis [15], RPLN involvement is rare. To date, the largest cohort of differentiated thyroid cancer (DTC) patients with RPLN metastases was reported by the Memorial Sloan Kettering group [10]. Their cohort consisted of 65 patients, with 42% managed surgically. In a meta‐analysis of 44 studies, including 145 thyroid cancer patients with RPLN metastases, 69% were treated surgically, while 19% underwent observation and 12% received nonsurgical therapies [11]. Herein, we present the largest single‐institution cohort to date of thyroid cancer patients with RPLN metastases, reporting their natural history, treatment approaches, and outcomes.

## 2 Materials and Methods

### 2.1 Study Population

**FIGURE 1:**
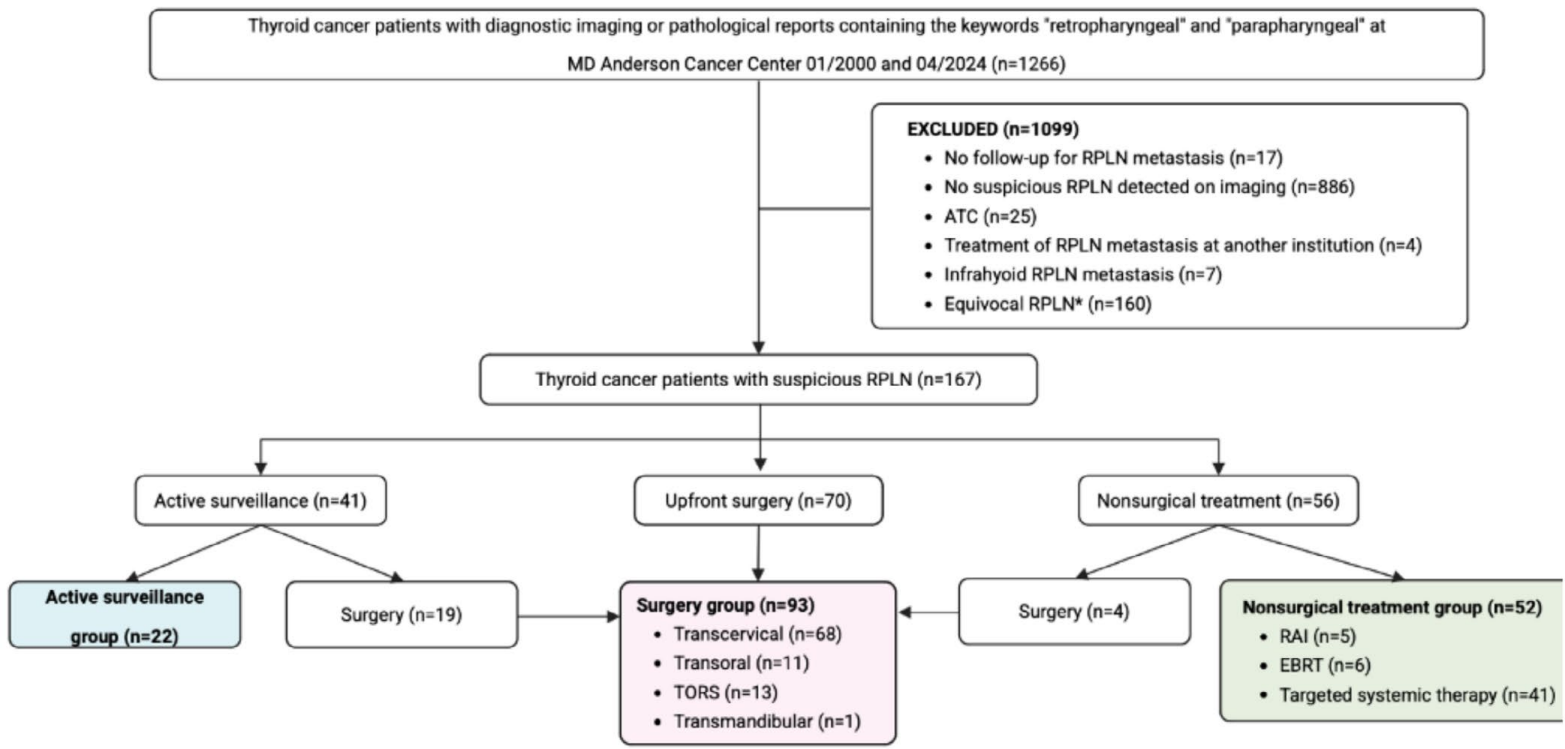
Flow diagram for study cohorts. *Equivocal RPLNs included patients with suspicious RPLNs in initial imaging that either resolved without treatment, were deemed non‐suspicious, or still equivocal after a period of active surveillance with follow‐up imaging, or had benign histopathological confirmation. ATC, anaplastic thyroid cancer; EBRT, external beam radiation therapy; RAI, radioactive iodine; RPLN, retropharyngeal lymph node; TORS, transoral robotic surgery. [Color figure can be viewed at wileyonlinelibrary.com]

This IRB‐approved (PA14‐1082) retrospective cohort study reviewed consecutive patients with non‐anaplastic thyroid cancer and RPLN metastases at the University of Texas MD Anderson Cancer Center (MDACC) between January 1st, 2000 and April 1st, 2024. Thyroid cancer patients were initially identified using the International Classification of Diseases (ICD) code for malignant neoplasms of the thyroid gland (C73). Pathology and diagnostic imaging reports, including CT (computed tomography) and PET (positron emission tomography)/CT, were subsequently queried for the terms “retropharyngeal” or “parapharyngeal.” RPLNs were defined as lymph nodes located within the suprahyoid RPS. Patients were excluded if they: (1) were diagnosed with RPLN metastasis but had no subsequent follow‐up at our institution; (2) had no RPLN metastasis, including patients whose imaging reports described RPLNs as “prominent,” “nonsuspicious,” “reactive,” “benign,” “nonspecific,” “without concerning features,” or “enlarged”; (3) had anaplastic thyroid cancer (ATC); (4) received treatment for RPLN metastasis at an outside institution; (5) had infrahyoid RPLN metastasis; or (6) had equivocal RPLN findings (Figure 1). Equivocal RPLNs included patients with suspicious RPLNs on initial imaging that either resolved without treatment, or were deemed non‐suspicious, or remained equivocal after a period of active surveillance (AS) with follow‐up imaging, or had benign histopathological confirmation.

A RPLN was considered metastatic from thyroid cancer if imaging reports included terms such as “suspicious,” “concerning,” “consistent with metastasis,” “presumably metastasis,” “suspected metastasis,” “possible metastasis,” “pathologic,” or “abnormal.” Patients with RPLN metastasis were divided into three groups for analysis: (1) the AS group, defined as patients with at least 6 months of observation alone for their RPLN metastasis; (2) the surgery group, including patients who underwent upfront surgery or AS/nonsurgical management followed by surgery; and (3) the nonsurgical treatment group, defined as patients treated with radioactive iodine (RAI), external beam radiation therapy (EBRT), systemic targeted therapy, or a combination of these modalities. All patients in the nonsurgical treatment group with RPLN metastases who were treated with RAI demonstrated significant RAI uptake on whole‐body scintigraphy (WBS).

### 2.2 Outcomes and Study Assessments

The primary outcomes of this study were: (1) the pretreatment size of RPLNs, defined by the greatest/longest axial dimension on imaging; (2) the growth rate of RPLNs during AS, calculated as the increase in the greatest/longest axial dimension per year; and (3) surgical outcomes, including operative approaches, postoperative complications, functional outcomes (dysphagia, hypernasality, and velopharyngeal insufficiency), and recurrence‐free survival (RFS). RFS was defined as the time from surgery to documented structural recurrence in the RPS on cross‐sectional imaging. Patients without recurrence were censored at the date of last follow‐up if alive, or at the date of death if deceased.

### 2.3 Statistical Analyses

Continuous variables are presented as medians and ranges, while categorical variables are presented as frequencies and percentages. The Kaplan–Meier method was used to analyze the RFS endpoint. Statistical analysis was performed with IBM SPSS Statistics for Macintosh, Version 29 (Armonk, NY).

## 3 Results

Between January 1st, 2000, and April 1st, 2024, a total of 1266 thyroid cancer patients with the terms “retropharyngeal” or “parapharyngeal” in their diagnostic imaging or pathology reports were evaluated at MDACC (Figure 1). Of these, 167 patients met the study's inclusion criteria and were divided into three groups: 22 patients in the AS group (13%), 93 in the surgery group (56%), and 52 in the nonsurgical treatment group (31%). Among the 167 patients (Table 1), 56% were male, with a median age of 47 years (range: 5–75) at thyroid cancer diagnosis and 52 years (range: 5–84) at RPLN metastasis diagnosis. Histologic subtypes included papillary thyroid cancer (PTC) (73%), medullary thyroid cancer (MTC) (21%), poorly differentiated thyroid cancer (PDTC) (4%), follicular thyroid cancer (FTC) (1%), and oncocytic thyroid cancer (OTC) (1%). The majority of RPLN metastases (71%) were diagnosed at the time of thyroid cancer recurrence, with CT being the primary imaging modality for detection in 90% of cases (Table 2). Biopsy was not performed in most cases (93%), and RPLN metastases were ipsilateral to the primary thyroid tumor in about half (51%) of patients. At the time of RPLN metastasis diagnosis, 8% had isolated RPLN involvement, 74% had concurrent locoregional disease, 6% had distant metastases, and 12% had both concurrent locoregional and distant disease. For patients diagnosed with thyroid cancer recurrence in RPLN (*n* = 118), the median time from initial thyroid cancer management to diagnosis of RPLN metastasis was 3.8 years (range: 0–51). At the time of RPLN metastasis diagnosis, the median size of the RPLN was 1.2 cm (range: 0.4–4.2), and the median follow‐up duration thereafter was 4.7 years (range: 0–20). For all patients who underwent AS, whether without subsequent treatment (*n* = 22) or followed by surgery (*n* = 19) or nonsurgical therapy (*n* = 19), the median annual growth in greatest/longest diameter of RPLNs was 10% (range: 0–138) or 0.09 cm (range: 0–1.4).

**TABLE 1:** Baseline demographic and tumor characteristics of 167 patients with suprahyoid retropharyngeal lymph node metastasis.

	Active surveillance (*n* = 22)	Surgery (*n* = 93)	Nonsurgical treatment (*n* = 52)	Total (*n* = 167)
Sex, *n* (%)				
Male	13 (59)	54 (58)	27 (52)	94 (56)
Female	9 (41)	39 (42)	25 (48)	73 (44)
Median age at thyroid cancer diagnosis, years (range)	43 (12–75)	44 (5–70)	53 (11–72)	47 (5–75)
Histology, *n* (%)				
PTC	19 (86)	75 (81)	28 (54)	122 (73)
FTC	0 (0)	1 (1)	1 (2)	2 (1)
OTC	0 (0)	1 (1)	1 (2)	2 (1)
MTC	3 (14)	12 (13)	20 (38)	35 (21)
PDTC	0 (0)	4 (4)	2 (4)	6 (4)
AJCC 8th edition stage, *n* (%)				
I	13 (59)	63 (68)	18 (35)	94 (56)
II	4 (18)	10 (11)	6 (11)	20 (12)
III	3 (13)	6 (6)	3 (6)	12 (7)
IVa	1 (5)	8 (9)	11 (21)	20 (12)
IVb	0 (0)	0 (0)	2 (4)	2 (1)
IVc	1 (5)	1 (1)	4 (8)	6 (4)
Unknown^a^	0 (0)	5 (5)	8 (15)	13 (8)

Abbreviations: AJCC, American Joint Committee on Cancer; FTC, follicular thyroid cancer; MTC, medullary thyroid cancer; *n*, number; OTC, oncocytic thyroid cancer; PDTC, poorly differentiated thyroid cancer; PTC, papillary thyroid cancer.

^a^ Patients initially treated elsewhere, without available staging data.

**TABLE 2:** Suprahyoid retropharyngeal lymph node metastasis characteristics.

	Active surveillance (*n* = 22)	Surgery (*n* = 93)	Nonsurgical treatment (*n* = 52)	Total (*n* = 167)
Median age at RPLN metastasis diagnosis, years (range)	49 (21–84)	49 (5–73)	60 (14–81)	52 (5–84)
RPLN metastasis diagnosis, *n* (%)				
At initial presentation	4 (18)	35 (38)	10 (19)	49 (29)
Recurrence	18 (82)	58 (62)	42 (81)	118 (71)
Imaging modalities for diagnosis, *n* (%)				
CT	21 (96)	80 (86)	49 (94)	150 (90)
MRI	0 (0)	5 (5)	0 (0)	5 (3)
PET/CT	1 (4)	8 (9)	3 (6)	12 (7)
RPLN metastasis location, *n* (%)				
Ipsilateral	12 (55)	51 (54)	22 (42)	85 (51)
Contralateral	4 (18)	6 (7)	3 (6)	13 (8)
Bilateral	1 (4)	6 (7)	8 (15)	15 (9)
NE^a^	5 (23)	30 (32)	19 (37)	54 (32)
Concurrent disease at RPLN metastasis diagnosis, *n* (%)				
None	6 (27)	6 (7)	2 (4)	14 (8)
Locoregional	14 (63)	79 (85)	30 (58)	123 (74)
Distant	1 (5)	3 (3)	6 (11)	10 (6)
Both locoregional and distant	1 (5)	5 (5)	14 (27)	20 (12)

Abbreviations: CT, computed tomography; MRI, magnetic resonance imaging; *n*, number; NE, not evaluable; PET, positron emission tomography; RPLN, retropharyngeal lymph node.

^a^ Bilateral primary tumor, or primary tumor location from initial surgery unavailable.

Patients were divided by thyroid cancer subtype, which included DTC (PTC, OTC, and FTC) (*n* = 126), MTC (*n* = 35), PDTC (*n* = 6). At the time of RPLN metastasis diagnosis, the median size of the RPLN was 1.3 cm (range: 0.4–4.2) for DTC, 1.1 cm (range: 0.6–3.4) for MTC, and 1.3 cm (range: 0.7–2.4) for PDTC. Among all patients who underwent AS (*n* = 60), 40 had DTC, 18 had MTC, and 2 had PDTC. For DTC, MTC, and PDTC patients, respectively, the median annual growth in the greatest/longest diameter of RPLNs was 5% (range: 0–138), 12% (range: 0–107), and 42% (range: 37–48), corresponding to an absolute growth of 0.04 cm (range: 0–1.4), 0.13 cm (range: 0–1), and 0.38 cm (range: 0.3–0.5).

### 3.1 Active Surveillance Group

Among the 22 patients in the AS group, the median initial size of RPLN metastasis was 0.9 cm (range: 0.4–3.0). After a median AS period of 2.3 years (range: 0.4–12.7), the median size of RPLNs increased to 1.1 cm (range: 0.6–3.0), representing a median annual growth rate of 3% (range: 0–56) or 0.02 cm (range: 0–0.7).

### 3.2 Surgery Group

Among the 93 patients included in the surgery group, 70 underwent upfront surgery, 19 underwent AS for at least 6 months prior to surgery, and 4 received targeted systemic therapy before ultimately undergoing surgery. Among the 19 patients who underwent AS prior to surgery, the median duration of AS was 2.2 years (range: 0.5–12.7), with a median RPLN metastasis growth of 11%/year (range: 0–138) or 0.15 cm/year (range: 0–1.4). Prior to RPLN dissection, the median RPLN metastasis size was 1.7 cm (range: 0.5–4.2). The surgical approach to the suprahyoid RPS was transcervical in 73% of cases, transoral in 12%, transoral robotic surgery (TORS) in 14%, and transmandibular in 1% (Table 3).

**TABLE 3:** Surgical cohort characteristics.

	Transcervical (*n* = 68)	Transoral (*n* = 11)	TORS (*n* = 13)	Transmandibular (*n* = 1)	Total (*n* = 93)
Treatment prior to RPLN dissection, *n* (%)					
Surgery	43 (63)	9 (82)	13 (100)	1 (100)	66 (71)
RAI	27 (40)	8 (73)	8 (62)	1 (100)	44 (47)
EBRT	5 (7)	2 (18)	2 (15)	0 (0)	9 (10)
Targeted systemic treatment	2 (3)	0 (0)	2 (15)	0 (0)	4 (4)
Concurrent surgery, *n* (%)					
No	4 (6)	5 (45)	11 (85)	0 (100)	20 (22)
Central neck dissection	48 (71)	5 (45)	0 (0)	1 (100)	54 (58)
Lateral neck dissection	61 (90)	6 (55)	2 (15)	1 (100)	70 (75)
Total thyroidectomy	25 (37)	2 (18)	0 (0)	0 (0)	27 (29)
Completion thyroidectomy	2 (3)	1 (9)	0 (0)	0 (0)	3 (3)
Tracheostomy	1 (1)	0 (0)	0 (0)	0 (0)	1 (1)
Laryngectomy^a^	4 (6)	0 (0)	0 (0)	0 (0)	4 (4)
Median size prior to surgery, cm (range)	1.6 (0.5–4)	1.5 (1–3.4)	2.5 (1–4.2)	2.4 (2.4–2.4)	1.7 (0.5–4.2)
Extracapsular spread, *n* (%)	41 (60)	6 (55)	11 (85)	1 (100)	59 (63)
Histology, *n* (%)					
DTC^b^	56 (82)	9 (82)	11 (85)	1 (100)	77 (83)
MTC	9 (13)	1 (9)	2 (15)	0 (100)	12 (13)
PDTC	3 (4)	1 (9)	0 (0)	0 (100)	4 (4)
Postoperative complications^c^, *n* (%)					
Intraoral wound dehiscence	—	0 (0)	2 (15)	—	2 (8)
Horner syndrome	1 (1)	0 (0)	0 (0)	0 (0)	1 (1)
First bite syndrome	1 (1)	0 (0)	0 (0)	0 (0)	1 (1)
Hypoglossal nerve palsy	2 (3)	0 (0)	0 (0)	1 (100)	3 (3)
Lingual edema	—	0 (0)	1 (8)	—	1 (4)
Transoral bleeding	—	0 (0)	3 (23)	—	3 (13)
Pharyngotomy	2 (3)	—	—	0 (0)	2 (3)
RPLN in surgical specimen, median (range)					
Positive	1 (1–3)	1 (1–1)	1 (1–2)	1 (1–1)	1 (1–3)
Total	1 (1–5)	1 (1–2)	1 (1–2)	1 (1–1)	1 (1–5)
Postoperative treatment, *n* (%)					
RAI	19 (28)	2 (18)	4 (31)	0 (0)	25 (27)
EBRT	5 (7)	0 (0)	0 (0)	0 (0)	5 (5)
Targeted therapy	1 (1)	0 (0)	0 (0)	0 (0)	1 (1)
Recurrence of RPLN metastasis, *n* (%)	3 (4)	3 (27)	5 (38)	0 (0)	11 (12)

Abbreviations: DTC, differentiated thyroid cancer; EBRT, external beam radiation therapy; MTC, medullary thyroid cancer; *n*, number; PDTC, poorly differentiated thyroid cancer; RAI, radioactive iodine; RPLN, retropharyngeal lymph node; TORS, transoral robotic surgery.

^a^ Three patients underwent total laryngectomy and one partial laryngectomy.

^b^ DTC includes PTC, FTC, and OTC.

^c^ Reported complications are limited to those associated with RPLN dissection.

At the time of RPLN metastasis diagnosis, 90% had concurrent locoregional disease; and more precisely, 74% had concurrent ipsilateral lateral neck disease. Concurrent surgery with RPLN dissection was performed in most cases (78%), including lateral neck dissection (75%), central neck dissection (58%), and total thyroidectomy (29%). Among patients who underwent a transcervical approach (*n* = 68), postoperative complications included permanent Horner's syndrome in 1 patient (1%), transient hypoglossal nerve palsy in 2 patients (3%), transient first bite syndrome in 1 patient (1%), and inadvertent pharyngotomy in 2 patients (3%). Among patients who underwent a transoral or TORS approach (*n* = 24), perioperative transoral bleeding occurred in 3 patients (13%), intraoral wound dehiscence in 2 patients (8%), and lingual edema in 1 patient (4%). Perioperative bleeding events included one patient who returned to OR on postoperative day 10 for intraoral cauterization, one patient who had interventional radiology embolization of the superior pharyngeal artery on postoperative day 3, and one patient who had intraoperative carotid artery injury with carotid stent placement.

**FIGURE 2:**
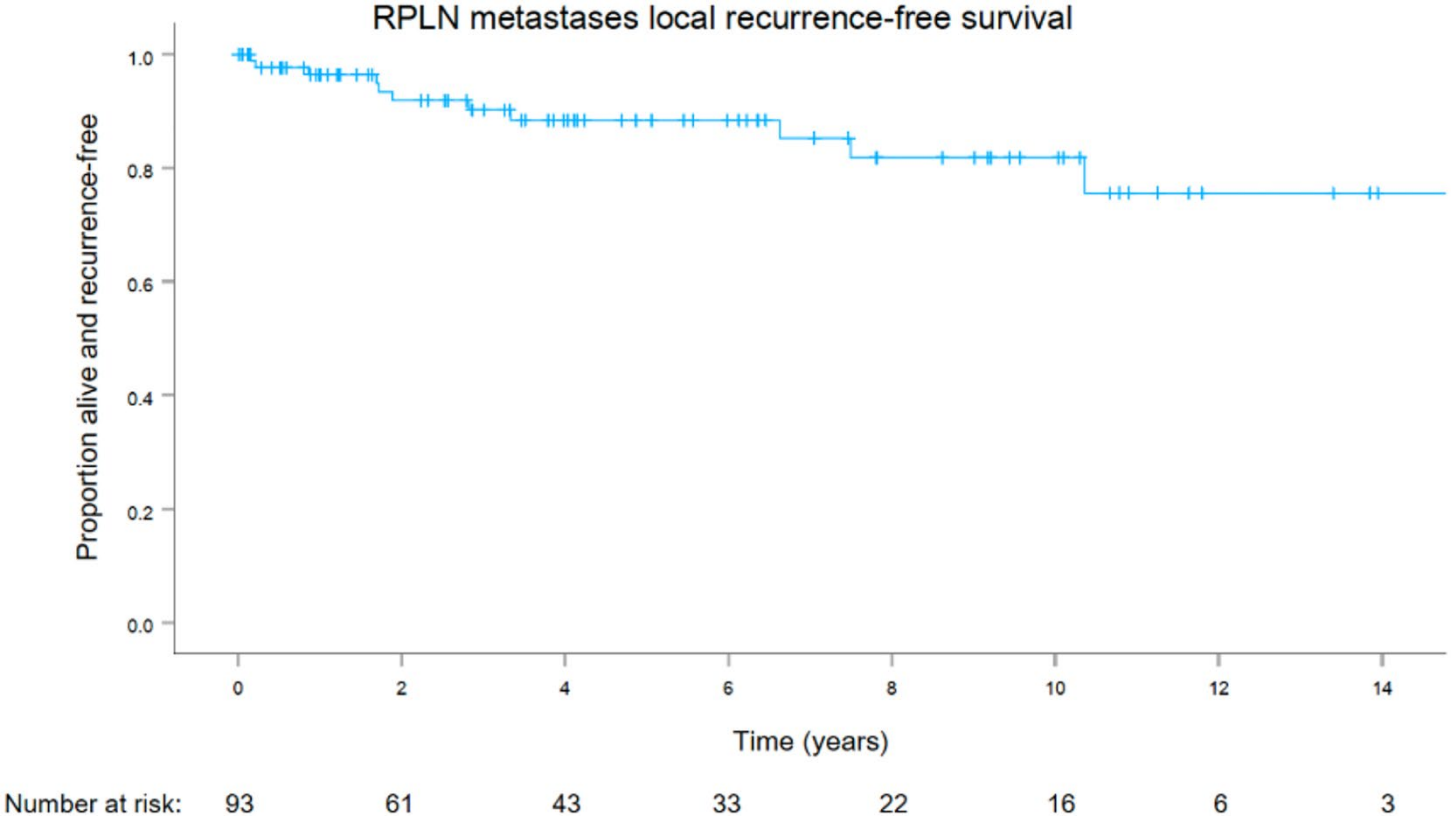
RPLN metastases local recurrence‐free survival after surgery. [Color figure can be viewed at wileyonlinelibrary.com]

In the RPLN surgical specimens, the median number of positive RPLNs was 1 (range: 1–3), and the median total number of RPLNs identified was 1 (range: 1–5). Eleven patients (12%) ultimately experienced RPLN metastasis recurrence, with median RFS not reached (Figure 2). The 5‐year and 10‐year RPLN metastasis local RFS were 88% [CI 80.7–96.1] and 82% [CI 70.5–93.1], respectively.

Postoperative functional assessment is reported in 87 patients (Table 4). Six patients who underwent laryngectomy/tracheostomy were excluded, but other high‐risk subgroups were included: patients with preoperative vocal cord paralysis (*n* = 20), those with intentional RLN (recurrent laryngeal nerve) sacrifice during concurrent surgical resection (*n* = 4), and patients with a history of neck EBRT (*n* = 9). Postoperatively, 11% of patients required temporary nasogastric feeding tube placement, with a median duration of 4 days (range: 1–41). More precisely, six patients had a feeding tube placed at the time of surgery, while four patients required feeding tube placement in the immediate postoperative period due to poor oral intake. Additionally, two patients (2%) who initially required a nasogastric feeding tube later underwent gastrostomy tube placement. Both of these patients had significant and invasive retroesophageal disease in addition to suprahyoid RPS involvement.

**TABLE 4:** Functional outcomes following retropharyngeal lymph node (RPLN) dissection.

	Surgery group (*n* = 87)^a^, ^b^
Temporary postoperative feeding tube, *n* (%)	10 (11)
Permanent feeding tube, *n* (%)	2 (2)
Dysphagia, *n* (%)	
Preoperative	15 (17)
Immediate postoperative	41 (47)
> 3 months postoperative^c^	9 (11)
> 6 months postoperative^c^	8 (10)
Hypernasality, *n* (%)	
Immediate postoperative	6 (7)
> 3 months postoperative^c^	0 (0)
Velopharyngeal insufficiency, *n* (%)	
Immediate postoperative	8 (9)
> 3 months postoperative^c^	1 (1)

^a^ Patients with total or partial laryngectomy, or tracheostomy, were excluded (*n* = 6).

^b^ Twenty patients presented with preoperative vocal cord paralysis, four underwent recurrent laryngeal nerve sacrifice during RPLN dissection, and nine had a history of neck EBRT.

^c^ *n* = 83, as four patients did not have 3 and 6 month follow‐up visits.

In the first 3 months after surgery, 59% of patients were evaluated by a speech‐language pathologist (SLP) and 18% underwent modified barium swallow (MBS) assessment. Dysphagia was reviewed at four timepoints: preoperatively (17% dysphagia), in the acute postoperative period (47%), at approximately 3 months postoperatively (11%), and at approximately 6 months postoperatively (10%). Among the 8 patients (9%) with persistent dysphagia beyond 6 months, 2 had preoperative RLN palsy, while 2 others underwent concurrent RLN sacrifice during their surgery (unrelated to the RPLN dissection), and 1 received postoperative neck EBRT. Eight patients (9%) had temporary velopharyngeal insufficiency, with only one case (1%) persisting beyond 3 months postoperatively. All six patients (7%) with hypernasality had complete resolution at 3 months.

### 3.3 Nonsurgical Treatment Group

Among the 52 patients in the nonsurgical treatment group, the treatment distribution was as follows: 10% RAI, 11% EBRT, and 79% targeted systemic therapy. Nineteen patients underwent AS before treatment, demonstrating a median RPLN metastasis growth rate of 18%/year (range: 0–109) or 0.16 cm/year (range: 0–1) over a median follow‐up of 2.2 years (range: 0.6–14.6). Prior to the initiation of treatment, the median RPLN metastasis size was 1 cm (range: 0.5–2.8). Among the five patients treated with RAI, the median I‐131 dose was 152 mCi (range: 103–201). Among the six patients who received definitive EBRT, 50% of treatment plans included bilateral retropharyngeal fields, while 83% of treatment plans involved both the central/lateral neck and retropharyngeal regions. The median radiation dose was 63 Gy (range: 27–66). In all 41 patients treated with targeted systemic therapy, treatment was initiated for RPLN metastasis in the context of concurrent locoregional or distant disease. Targeted systemic therapies included BRAF/MEK inhibitors (*n* = 16), multikinase inhibitors (*n* = 16), selective RET inhibitors (*n* = 8), and mTOR inhibitors (*n* = 1).

## 4 Discussion

We present the largest reported series to date on the management of RPLN metastasis in thyroid cancer. Although the existing literature on RPLN metastases has focused predominantly on DTC, our cohort includes a significant proportion of patients with MTC (21%).

Transcervical ultrasonography (US), a noninvasive and cost‐effective modality, is the first‐line imaging technique for nodal basin evaluation in thyroid cancer. However, due to its deep anatomical location, RPLN metastases are typically poorly visualized on US, necessitating alternative imaging (CT, magnetic resonance imaging [MRI], PET/CT, and SPECT [single photon emission computed tomography]‐CT on RAI scan) for accurate detection. In our cohort, all RPLN metastases were detected using one of these modalities. Internal calcifications and necrotic changes represent key radiographic features of RPLN metastasis. Although no formal consensus exists regarding size criteria, a minimum axial diameter of 6 mm has been proposed as the most accurate threshold for identifying metastatic RPLNs [16]. Another study evaluated the size of normal RPLNs on MRI across different age groups and found that the maximal axial diameter was 6.6 ± 1.7 mm in patients aged 20–38 years and 5.3 ± 1.6 mm in those aged 42–74 years [17]. While histopathological confirmation remains the diagnostic gold standard in oncology, RPLN biopsy presents unique technical challenges due to the surrounding neurovascular anatomy and difficulty in accessing this area [18, 19], explaining why only 7% of patients with RPLN metastases in our series underwent biopsy confirmation.

The low rate of RPLN metastasis at the time of initial thyroid cancer diagnosis in this study (29%) corroborates existing data that RPLN involvement often manifests as residual or recurrent disease in most patients (75%–81%) [10, 11]. Among surgical patients with RPLN metastasis, given the high rate of concurrent locoregional disease (90%), we recommend evaluating the RPLN basin before proceeding with therapeutic lateral neck dissection for either initial or recurrent metastatic thyroid cancer. This recommendation is supported by our finding that a majority (74%) of RPLN metastases occurred concurrently with ipsilateral lateral neck disease. For RPLN metastasis ≥ 1 cm, transcervical excision at the time of lateral neck dissection is recommended. The surgical technique for transcervical resection of RPLN metastasis from thyroid cancer has been previously described [20]. The management of RPLN metastasis without concurrent lateral neck disease warrants additional consideration, as the risks and benefits of intervention must be carefully weighed. When no concurrent surgery is necessary, we recommend evaluation for transcervical resection or TORS. In our study, 85% of TORS cases were performed in patients with isolated retropharyngeal disease, with median tumor size 2.5 cm at the time of surgery.

Our experience demonstrates that AS represents a safe strategy in select cases, with observed median growth rates of 10%/year or 0.09 cm/year for the overall study cohort. Growth of RPLN metastases under AS often paradoxically facilitates subsequent surgical resection by improving intraoperative palpation and visualization. RPLN metastases which are < 1 cm may undergo AS, as many of these may never need to be treated, and lymph nodes < 1 cm in the RPS can be technically challenging to palpate and resect through a limited surgical window. As such, only four patients in the current study had surgical excision of retropharyngeal lymph nodes < 1 cm, all of whom were undergoing concomitant lateral neck surgery for lateral neck disease. For patients undergoing an ipsilateral lateral neck dissection, RPLN metastases (especially those ≥ 1 cm) should be considered for transcervical excision at the time of lateral neck dissection.

In our study, 73% of RPLN dissections were performed via a transcervical approach, and 90% of dissections were performed concurrently with a lateral neck dissection for concurrent lateral neck disease. On the other hand, 14% of RPLN dissections were performed via TORS, and only 15% of these cases had concurrent lateral neck surgery, suggesting that this technique is often utilized in patients who are not undergoing concurrent lateral neck dissection. This is consistent with Harries et al. [10], who reported transcervical and TORS approaches in 70% and 22% of cases, respectively. Similarly, a systematic review by Sandler et al. [11] found that transcervical, transoral, TORS, and transcervical‐transmandibular approaches were used in 65%, 28%, 4%, and 3% of cases, respectively.

The transmandibular approach, while historically utilized, is now very rarely indicated. In our study, only one patient with partial carotid encasement underwent RPLN dissection via this approach in 2010. Given its higher morbidity, this technique should generally be avoided for thyroid cancer patients, as metastases are generally able to be excised either transcervically or transorally. The conventional transoral approach is not currently used at our institution, with all cases in this study undergoing this technique prior to 2011. In contemporary practice, transoral procedures are generally robotic‐assisted or endoscopic‐assisted, as robotic platforms offer superior surgical field visualization and enable more precise resection [21]. Three patients in the TORS group had significant perioperative or postoperative bleeding warranting acute intervention, representing a significant perioperative risk that should be discussed prior to surgery.

Overall, 11 (12%) patients had recurrence in the RPS following RPLN dissection, with recurrence rates of 4% and 38% among the transcervical and TORS groups, respectively. It should be noted that these two groups are not directly comparable, as the patients who underwent TORS generally had larger tumors and higher rates of extracapsular nodal extension. Additionally, all patients in the TORS group had a previous surgery, while only 63% of patients in the transcervical group had previous surgery, suggesting potential differing tumor biology between the two groups.

Postoperative dysphagia occurred in 47% of patients immediately following surgery, an expected consequence of dissection along the superior and middle pharyngeal constrictor muscles. Eleven percent of patients ultimately required temporary feeding tube placement, which was removed after a median of 4 days (range: 1–41), indicating that most patients were able to maintain functional oral intake despite dysphagia symptoms. Only 2 patients (2%) ultimately required gastrostomy tube placement postoperatively, both of whom had extensive concurrent esophageal and retroesophageal disease in the neck along with RPLN metastasis, such that the permanent feeding tube status was not primarily related to the RPLN dissection in these patients. In comparison, in a small case series of 12 patients who had RPLN dissection for thyroid cancer, 58% had dysphagia after surgery [14].

Limitations of this study include its retrospective design and the potential selection bias inherent to a tertiary cancer center referral population. Despite being the gold standard for RPLN metastasis, histopathological confirmation was not routinely performed for patients in the AS and nonsurgical treatment groups. Additionally, dysphagia was evaluated subjectively without the use of a validated instrument, which may introduce reporting bias. Finally, the incidence of RPLN metastases may be underreported in this study due to limitations in imaging technology to differentiate benign from malignant RPLN. Despite these limitations, this large cohort provides valuable insights into the natural history and management of RPLN metastasis in thyroid cancer patients.

In conclusion, RPLN metastases from thyroid cancer typically grow slowly, making AS an appropriate management strategy for small‐volume disease, especially when a patient is not already undergoing concurrent lateral neck surgery. For patients with RPLN metastasis ≥ 1 cm, RPLN dissection should generally be performed via a transcervical approach in cases with concurrent lateral neck disease. For isolated RPLN metastasis, either a transcervical approach or TORS may be considered. Patients should be counseled on the likelihood of temporary dysphagia and the possibility of temporary velopharyngeal insufficiency and hypernasality, although significant long‐term morbidity is uncommon.

## Author Contributions

**I.F.:** data curation, conceptualization, methodology, formal analysis, writing – original draft preparation, writing – reviewing and editing. **A.V.B.:** data curation. **S.H.:** writing – reviewing and editing. **P.I.:** writing – reviewing and editing. **N.L.B.:** writing – reviewing and editing. **M.E.C.:** writing – reviewing and editing. **R.D.:** writing – reviewing and editing. **M.I.H.:** writing – reviewing and editing. **S.G.W.:** writing – reviewing and editing. **U.N.B.:** data curation. **M.D.W.:** writing – reviewing and editing. **M.M.:** writing – reviewing and editing. **M.G.M.:** writing – reviewing and editing. **N.D.G.:** writing – reviewing and editing. **R.P.G.:** writing – reviewing and editing. **N.D.P.:** writing – reviewing and editing. **V.B.:** writing – reviewing and editing. **J.R.W.:** writing – reviewing and editing. **A.M.:** writing – reviewing and editing. **M.E.Z.:** data curation, conceptualization, methodology, writing – original draft preparation, writing – reviewing and editing.

## Ethics Statement

All procedures were conducted in accordance with the institutional ethics committee's standards for human research.

## Conflicts of Interest

N.L.B. reports research funding from Eisai and personal consulting fees from Eisai and Eli Lilly. M.E.C. has received consulting fees from Bayer, Exelixis, Lilly, Novartis, and research funding from Merck, Genentech, Eisai, Exelixis. R.D. reports grants from Exelixis, Eisai, Merck, and AstraZeneca, personal fees from Bayer and Exelixis. M.I.H. reports research funding from Lilly and personal fees from Crispr therapeutics. S.G.W. reports personal fees from Bayer and consulting fees from Camurus. M.D.W. reports grants from Bayer and speaker fees from Springer Health. M.G.M. reports grants from Bayer pharmaceuticals for an educational grant and salary support from Siemens as part of an institutional development deal. A.M. reports research funding from Jazz Pharmaceuticals and Thryv Therapeutics Inc. M.E.Z. reports research funding from Merck, Eli Lilly, and Exelixis. The other authors declare no conflicts of interest.

## Data Availability

The data that support the findings of this study are available on request from the corresponding author. The data are not publicly available due to privacy or ethical restrictions.
